# Purification and properties of *S*-hydroxymethylglutathione dehydrogenase of *Paecilomyces variotii* no. 5, a formaldehyde-degrading fungus

**DOI:** 10.1186/2191-0855-2-32

**Published:** 2012-06-25

**Authors:** Ryohei Fukuda, Kazuhiro Nagahama, Kohsai Fukuda, Keisuke Ekino, Takuji Oka, Yoshiyuki Nomura

**Affiliations:** 1 Department of Applied Microbial Technology, Faculty of Biotechnology and Life Science, Sojo University, Ikeda 4-22-1, Nishi-ku, Kumamoto City, 860-0082, Japan

**Keywords:** *S*-hydroxymethylglutathione dehydrogenase, Enzyme purification, Formaldehyde metabolism, *Paecilomyces variotii*, SH enzyme

## Abstract

*S*-hydroxymethylglutathione dehydrogenase from *Paecilomyces variotii* No. 5 strain (NBRC 109023), isolated as a formaldehyde-degrading fungus, was purified by a procedure that included ammonium sulfate precipitation, DEAE-Sepharose and hydroxyapatite chromatography and isoelectrofocusing. Approximately 122-fold purification was achieved with a yield of 10.5%. The enzyme preparation was homogeneous as judged by sodium dodecyl polyacrylamide gel electrophoresis (SDS-PAGE). The molecular mass of the purified enzyme was estimated to be 49 kDa by SDS-PAGE and gel filtration, suggesting that it is a monomer. Enzyme activity was optimal at pH 8.0 and was stable in the range of pH 7.0–10. The optimum temperature for activity was 40°C and the enzyme was stable up to 40°C. The isoelectric point was pH 5.8. Substrate specificity was very high for formaldehyde. Besides formaldehyde, the only aldehyde or alcohol tested that served as a substrate was pyruvaldehyde. Enzyme activity was enhanced by several divalent cations such as Mn^2+^ (179%), Ba^2+^ (132%), and Ca^2+^ (112%) but was completely inhibited by Ni^2+^, Fe^3+^, Hg^2+^, p-chloromercuribenzoate (PCMB) and cuprizone. Inactivation of the enzyme by sulfhydryl reagents (Hg^2+^ and PCMB) indicated that the sulfhydryl group of the enzyme is essential for catalytic activity.

## Introduction

Formaldehyde is a ubiquitous compound that is a product of biological sources (from photooxidation of atmospheric hydrocarbons) ([Levy 1971]; [Zimmerman et al. 1978]) and environmental sources (emissions from industrial processes) ([Ando 1998]). An advanced technology for potable water pretreatment includes ozonation, during which formaldehyde is generated as a result of the reaction of ozone with traces of humus ([Schechter and Singer 1995]). Formaldehyde acts as disinfectant at concentrations as low as 0.1%. Therefore, it is used for room sterilization, viscosity stabilization and preservation of adhesives made from starch and for preservation of experimental specimens. Formaldehyde is a highly toxic compound due to nonspecific reactivity with proteins and nucleic acids ([Grafstrom et al. 1983]), so it is an environmental pollutant.

To address the problem of formaldehyde pollution, we attempted to isolate a microorganism that can degrade formaldehyde. We isolated a fungus that can degrade concentrations of formaldehyde as high as 2.4%. The fungus belongs to the genus *Paecilomyces* ([Iwahara et al. 2002]). After determining the DNA sequence of the 18 S ribosomal RNA gene of this fungus, we named it *Paecilomyces variotii* No. 5 (NBRC 109023).

Based on the nature of the electron acceptor, formaldehyde-oxidizing enzymes are divided into two groups, NAD(P)^+^-dependent and dye (cytochrome)-linked. The NAD(P)^+^-dependent enzymes are further subdivided based on the need for secondary cofactors, such as thiol compounds, tetrahydrofolate, methylene tetrahydromethanopterin, or modifier proteins ([Zahn et al. 2001]). The oxidation of formaldehyde in eukaryotic cells is mainly carried out by NAD^+^ and glutathione-dependent formaldehyde dehydrogenase ([Achkor et al. 2003]; [Koivusalo et al. 1989]).

NAD^+^ and glutathione-dependent formaldehyde dehydrogenase, called *S*-hydroxymethylglutathione (*S*-HMGSH) dehydrogenase, catalyzes the following reaction:

(1) $$S‐\text{HMGSH}+{\text{NAD}}^{+}⇄S‐\text{formylglutathione}+\text{NADH}+{\text{H}}^{+}$$

Where *S*-HMGSH is a nonenzymatically (Uotila and Koivusalo [1974]) or enzymatically formed adduct of glutathione and formaldehyde ([Goenrich et al. 2002]). *S*-formylglutathione is oxidized further via formate to carbon dioxide. The formation of *S*-HMGSH from formaldehyde and glutathione is a central reaction in the consumption of the cytotoxic formaldehyde in some methylotrophic bacteria as well as in many other organisms.

Though many studies have reported on purification and characterization of *S*-HMGSH dehydrogenase produced by microorganisms, most were on enzymes from bacteria and yeasts ([Demkiv et al. 2007]; [Fernandez et al. 1995]; [Gutheil et al. 1992]; [Patel et al. 1983]; [Ras et al. 1995]; [Schutte et al. 1976]). There have been no reports on purified *S*-HMGSH dehydrogenase from fungi.

To investigate the mechanism of degradation of high concentrations of formaldehyde by *P. variotii* NBRC 109023, we attempted to purify *S*-HMGSH dehydrogenase, a key enzyme of detoxification in eukaryotic organisms, and succeeded in obtaining an electrophoretically homogenous preparation of the enzyme. Alcohol oxidase could also oxidize formaldehyde ([Sahm 1975]), and alcohol oxidase from *P. variotii* was purified ([Kondo et al. 2008]). However, this enzyme is not *S*-HMGSH dehydrogenase. To our knowledge, this article is the first report on purification of *S*-HMGSH dehydrogenase from a fungus. Herein, we describe the purification and properties of *S*-HMGSH dehydrogenase from *P. variotii* NBRC 109023.

## Materials and methods

### Chemicals

Malt extract was purchased from Oriental Yeast Co., Ltd. (Tokyo, Japan). Formaldehyde solution (37% (w/v), special grade), yeast extract D-3, polypeptone, carboxymethyl cellulose sodium salt, glutathione, NAD^+^ and all other chemicals were purchased from Wako Pure Chemical Industries, Ltd. (Osaka, Japan).

### Microorganism, media and culture conditions

*P. variotii* NBRC 109023, which was isolated from soil and can degrade a high concentration of formaldehyde (2.4%) was used. Table 1 shows the composition of the media used for its culture.

**Table 1:** Composition of media

**Media**	**Components of media (%**^a^**)**						
	**Glucose**	**Yeast extract**	**Malt extract**	**Polypeptone**	**Formaldehyde**	**CMC**^b^	**Agar**
Basal medium	0.5	0.3	0.3	1.0			
Medium for stock culture	0.5	0.3	0.3	1.0	0.5		1.5
Medium for seed culture	0.5	0.3	0.3	1.0	0.3	2.0	
Medium for enzyme production	0.5	0.3	0.3	1.0	1.0		

pH of media was adjusted at 7.0.

^a^ Percentage exhibits weight per volume.

^b^ Carboxymethyl cellulose sodium salt.

Stock cultures of *P. variotii* NBRC 109023 (5 mm × 5 mm) were inoculated into 300 ml Erlenmeyer flasks containing 100 ml of medium for seed culture and cultured on a rotary shaker (220 rpm) at 25°C for 5 days. In cultures for production of *S*-HMGSH dehydrogenase, 3-L shaking flasks containing 1 L of medium were inoculated with 1.5% (v/v) seed culture and cultured on a reciprocal shaker (120 strokes/min) at 25°C for 1 week.

### Preparation of crude enzyme

Shaking flasks containing medium for production of *S*-HMGSH dehydrogenase were inoculated and cultured on a reciprocal shaker at 25°C for 1 week. After cultivation, cell pellets were harvested and washed twice with 20 mM Tris–HCl buffer (pH 8.0). After centrifugation (21,000 × g for 20 min), the pellets were stored in the freezer (−30°C) before use. The frozen pellets (556 g) were suspended in 20 mM Tris–HCl buffer (pH 8.0). Sea sand (420–840 μm, 20–35 mesh, Wako Pure Chemical Industries, Ltd.) was added to the suspension. The pellets were ground in a mortar and pestle in a cold room (4°C). After cell disruption, cell debris was removed by centrifugation (21,000 × g for 20 min). The supernatant was used as crude enzyme solution.

### Purification of *S*-HMGSH dehydrogenase

The cell-free extract was salted out by a 30% saturated ammonium sulfate solution. After storage in an ice bath for 2 h, the resulting precipitate was removed by centrifugation (21,000 × g for 20 min), and the concentration of ammonium sulfate in the supernatant was brought to 80% saturation, the mixture being kept at 4°C for 2 h. The resulting precipitate was collected by centrifugation and dissolved in a small amount of 20 mM Tris–HCl buffer (pH 8.0). The enzyme solution was dialyzed against the same buffer at 4°C overnight. The dialyzed enzyme solution was applied to a column (3 cm i.d. × 25 cm length) of DEAE-Sepharose (Pharmacia, Uppsala, Sweden) equilibrated with the above buffer. After the column was washed with the same buffer, the adsorbed enzyme was eluted with an increasing linear gradient of NaCl from 0 to 0.2 M in the buffer. Active fractions were collected and applied to a column of hydroxyapatite (3.0 cm i.d. × 15 cm length) equilibrated with 20 mM Tris–HCl buffer (pH 8.0). The column was washed with the same buffer, and adsorbed enzyme was eluted using a linear gradient from 0 to 0.1 M phosphate buffer (pH 8.0). Active fractions were collected and dialyzed against 5 mM Tris–HCl buffer (pH 8.0). After dialysis, the enzyme solution was lyophilized. The lyophilized protein was dissolved with a small amount of solution containing 0.5 ml of 40% ampholyte (pH 3–10; Pharmacia) and 74.5 ml deionized water. Isoelectrofocusing was carried out by the method of [Matsuo and Horio (1967)] in a 110 ml electrophoresis column at 4°C for 3 days at 1 W.

### Measurement of enzyme activity

The activity of *S*-HMGSH dehydrogenase was assayed according to the method of [Kato (1990)] with a slight modification (see below). The activity was assayed at 35°C by the time and reductant-dependent formation of NADH from NAD^+^. The composition of the reaction mixture was as follows: 1.0 ml 50 mM Tris–HCl buffer (pH 8.0), 0.25 ml 120 mM glutathione, 0.25 ml 60 mM NAD^+^, 0.25 ml 60 mM formaldehyde (5.4 mM final concentration) and 1.0 ml deionized water. The reaction mixture was preincubated at 35°C for 5 min and the reaction was carried out by addition of 20 μl of the enzyme solution for 10 min (standard assay conditions). A blank test was performed with the same reaction mixture with formaldehyde omitted.

The increase in absorbance at 340 nm was followed against a blank using a Beckman DU-530 spectrophotometer (Beckman Coulter Inc., Brea, CA, USA) with a temperature-control module. The absorbance at 340 nm was recorded. One unit of enzyme activity was defined as the amount of enzyme catalyzing the formation of 1 μmol NADH per minute at 35°C.

In determining the optimum pH and pH range over which the enzyme was stable, the following buffer solutions were used: 50 mM citrate-NaOH buffer (pH 3–6), 50 mM phosphate buffer (pH 6–8), and 50 mM borate-NaOH buffer (pH 8–10). In determining enzyme stability at different pH values, the enzyme solution was kept at 25°C for 20 h and residual activity was measured under standard assay conditions. To determine stability at different temperatures, the enzyme solution was treated for 30 min and residual activity was measured under standard assay conditions.

In assessing the effects of metal ions and chemical compounds on the enzyme, the enzyme in 50 mM Tris–HCl buffer (pH 8.0) was treated with 1 mM of each compound (except EDTA; 10 mM) for 1 h at 30°C and residual activity was measured.

### Gel filtration

The molecular mass of the native enzyme was estimated by gel filtration on a TSK-gel G2000SW column (7.5 mm × 60 cm, Tosoh Corporation, Tokyo, Japan) equilibrated with 10 mM potassium phosphate buffer (pH 7.0) containing 0.1 M NaCl. The molecular mass was calibrated by comparing the retention time to a gel filtration standard (Serva Electrophoresis GmbH, Heidelberg, Germany) containing bovine serum albumin (67 kDa), ovalbumin (45 kDa), chymotrypsinogen A from bovine pancreas (25 kDa) and ribonuclease A from bovine pancreas (13.7 kDa)*.*

### Gel electrophoresis

Sodium dodecyl-polyacrylamide gel electrophoresis (SDS-PAGE) was done in a 12.5% polyacrylamide slab gel by the method of [Laemmli (1970)]. The molecular mass markers for SDS-PAGE were rabbit muscle phosphorylase b (97.0 kDa), bovine serum albumin (66.0 kDa), egg white ovalbumin (45.0 kDa), bovine carbonic anhydrase (30.0 kDa), and trypsin inhibitor (20.1 kDa) purchased from Amersham Biosciences (Uppsala, Sweden). The proteins were stained with Bio-Safe Coomassie Stain (Bio-Rad Laboratories, Inc., Hercules, CA, USA).

### Protein determination

Protein in samples was determined based on their absorbance at 280 nm and calculated using bovine serum albumin as the standard.

## Results

### Purification of *S*-HMGSH dehydrogenase

*S*-HMGSH dehydrogenase was purified from a cell-free extract of *P. variotii* NBRC 109023 by ammonium sulfate precipitation, DEAE-Sepharose chromatography, hydroxyapatite chromatography and isoelectrofocusing. Approximately 122-fold purification was achieved, with an overall yield of 10.5% from the cell-free extract. The details of the purification are summarized in Table 2.

**Table 2:** **Summary of purification of *S* -HMGSH dehydrogenase from *P. variotii* NBRC 109023**

	**Total protein**	**Total activity**	**Specific activity**	**Yield**	**Purification**
	**(mg)**	**(Units)**	**(U/mg)**	**(%)**	**(fold)**
Cell-free extract	10,820	23,312	2.2	100	1
Ammonium sulfate	1,539	11,080	7.2	47.5	3.3
DEAE-Sepharose	113	7,912	70	33.9	32
Hydroxyapatite	13.1	5,079	388	21.8	176
Lyophilization	10.0	2,503	250	10.7	114
Isoelectrofocusing	9.1	2,452	269	10.5	122

### Criteria for determining purity and molecular weight

**Figure 1:**
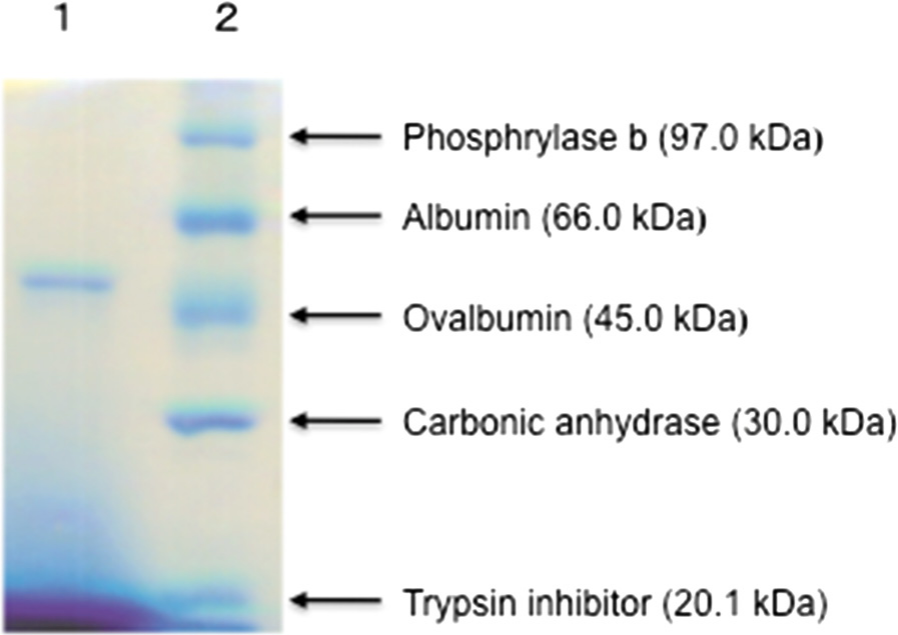
**SDS-PAGE of purified *S* -HMGSH dehydrogenase from *P. variotii* NBRC 109023.** Lane 1, purified enzyme. Lane 2, molecular mass markers. The protein band was stained with Coomassie Brilliant Blue.

The purity of the purified enzyme preparation was checked by SDS-PAGE. Figure 1 shows the electrophoretic pattern of a sample stained with Coomassie Brilliant Blue R 250; the purified enzyme gave a single band after electrophoresis. The molecular weight of the purified enzyme was estimated to be appropriately 49 kDa based on comparison with the mobility of marker proteins by SDS-PAGE. The molecular weight was also estimated to be 49 kDa as judged by gel filtration on TSK-gel G2000SW (data not shown), suggesting that it is a monomer. The isoelectric point of this enzyme was 5.8.

We attempted to determine the N-terminal amino acid of the purified enzyme using an Applied Biosystems 476 protein sequencer, but the N-terminus of this enzyme was blocked. Therefore, we used a protein N-terminal deblocking kit and pyroglutamate aminopeptidase (Takara Shuzo Co., Ltd., Kyoto Japan) to remove the formyl, pyroglutamyl, or acetyl group of the N-terminal amino acid, but unfortunately we could still not detect the N-terminal amino acid of *S*-HMGSH dehydrogenase from *P. variotii* NBRC 109023.

### Effects of pH and temperature on the activity and stability of the *S*-HMGSH dehydrogenase

**Figure 2:**
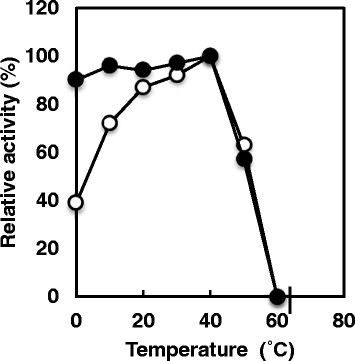
**Effects of temperature on activity and stability of purified *S* -HMGSH dehydrogenase.** The reaction was carried out under standard assay conditions described in the Materials and methods section, except for varying the temperature (open symbols). In experiments on stability, the enzyme was treated at various temperatures for 30 min in 50 mM phosphate buffer (pH 8.0) and the activity remaining was measured under standard assay conditions (closed symbols).

**Figure 3:**
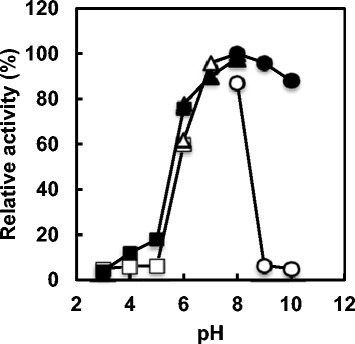
**Effects of pH on activity and stability of purified *S* -HMGSH dehydrogenase.** The following buffer solutions were used: 50 mM citrate-NaOH buffer (squares, pH 3–6), 50 mM phosphate buffer (triangles, pH 6–8) and 50 mM borate-NaOH buffer (circles, pH 8–10). The reaction was carried out at 35°C for 10 min at various pH values (open symbols). In pH stability experiments, the enzyme was treated at 25°C for 20 h at various pH values, and the activity remaining was measured under standard assay conditions (closed symbols).

The purified enzyme showed maximum activity at 40°C and was stable up to 40°C (Figure 2). At temperatures above 40°C, the enzyme activity declined sharply. The enzyme showed maximum activity at pH 8.0, and in the alkaline region, activity declined rapidly. In the narrow range of pH 7.0–10, the activity was stable (Figure 3).

### Substrate specificity of *S*-HMGSH dehydrogenase

The substrate specificity of *S*-HMGSH dehydrogenase was examined. Various substrates were added to the reaction mixture (5.4 mM final concentration) as mentioned in the Materials and methods. As shown in Table 3, *S*-HMGSH dehydrogenase from *P. variotii* NBRC 109023 has very high substrate specificity for formaldehyde. Besides formaldehyde, the only aldehyde or alcohol tested that served as a substrate was pyruvaldehyde (showing 26% the activity of formaldehyde).

**Table 3:** **Substrate specificity of *S* -HMGSH dehydrogenase**

**Substrate**	**Relative activity (%)**
Formaldehyde	100
Acetoaldehyde	0
Propylaldehyde	0
Butyraldehyde	0
Isobutylaldehyde	0
Oxalaldehyde	1>
Pyruvaldehyde	26
Methyl alcohol	0
Ethyl alcohol	0
Propyl alcohol	0

The reaction was carried out at pH 8.0 for 10 min.

Each substrate was 5.4 mM in a final concentration.

### Effects of various compounds on activity of *S*-HMGSH dehydrogenase

The effects of various compounds on enzyme activity were examined. As shown in Table 4, the enzyme activity was enhanced by several divalent cations such as Mn^2+^ (179%), Ba^2+^ (132%) and Ca^2+^ (112%). On the other hand, Ni^+2^, Fe^+3^, Hg^+2^, p-chloromercuribenzoate (PCMB) and cuprizone completely inhibited the activity. Inactivation of the enzyme by sulfhydryl reagents (Hg^2+^ and PCMB) indicated that the sulfhydryl group of this enzyme is essential for its catalytic activity.

**Table 4:** **Effects of various compounds on *S* -HMGSH dehydrogenase activity**

**Compound**	**Concentration**	**Remaining**
	**(mM)**	**activity (%)**
None		100
NiCl_2_	1	0
MnCl_2_	1	179
CoSO_4_	1	40
FeCl_2_	1	95
FeCl_3_	1	0
BaCl_2_	1	132
CaCl_2_	1	112
HgCl_2_	1	0
MgCl_2_	1	49
NaCl	1	84
EDTA	10	92
PCMB	1	0
Ellman reagent	1	68
PMSF	1	11
Cuprizone	1	0
Hydroxyl amine	1	60
Semicarbazide	1	67
Dithiothreitol	1	92

The enzyme in Tris–HCl buffer (pH 8.0) was treated by various compounds at 30°C for 1 hr and the reaction was carried out at pH 8.0 for 10 min.

## Discussion

We isolated a fungus that can degrade a concentration of formaldehyde as high as 2.4%. Based on the DNA sequence of 18S the ribosomal RNA gene of this fungus, we named it *P. variotii* NBRC 109023. To investigate the mechanism of degradation of high concentrations of formaldehyde by *P. variotii* NBRC 109023, we attempted to purify *S*-HMGSH dehydrogenase, a key enzyme of detoxification in eukaryotic organisms. *S*-HMGSH dehydrogenase was purified from a cell-free extract of *P. variotii* NBRC 109023 by ammonium sulfate precipitation, DEAE-Sepharose chromatography, hydroxyapatite chromatography and isoelectrofocusing. The purity of the purified enzyme preparation was checked by SDS-PAGE. The purified enzyme gave a single band after electrophoresis. The molecular weight of the purified enzyme was estimated to be approximately 49 kDa by SDS-PAGE and chromatography on TSK-gel G2000SW, suggesting that it is a monomer with an isoelectric point of 5.8.

*S*-HMGSH dehydrogenase is a dimeric enzyme with a 40-kDa subunit and is ubiquitous in eukaryotic organisms ([Uotila and Koivusalo 1989]). Table 5 compares *S*-HMGSH dehydrogenase from *P. variotii* NBRC 109023 and other organisms. Almost all *S*-HMGSH dehydrogenases are dimers with 40-kDa subunits, but *S*-HMGSH dehydrogenase from *P. variotii* NBRC 109023 is a 49-kDa monomer. We tried to get information for amino acid sequence of this enzyme, but we were unable to determine its N-terminal amino acid.

**Table 5:** **Comparison of the molecular weight of *S* -HMGSH dehydrogenase from *P. variotii* NBRC 109023 to those of other organisms**

**Organism**	**Molecular weight (kDa)**		**reference**
	**SDS-PAGE**	**Native**	
*Paracoccus denitrificans*	40	150	Ras et al. [1995]
*Escherichia coli*	n.r.	83	Gutheil et al. [1992]
		dimer	
*Candida boidini*	40	80	Schutte et al. [1976]
*Hansenula polymorpha*	40	dimer	
		(presumed)	Demkiv et al. [2007]
*Pichia* sp.	41	84	Patel et al. [1983]
Rat liver	41	41	Tsuboi et al. [1992]
*P. variotii* NBRC 109023	49	49	This study

n.r. means not reported.

The effects of pH and temperature on the activity and stability of the *S*-HMGSH dehydrogenase was investigated. The purified enzyme showed maximum activity at 40°C and was stable up to 40°C. At temperatures greater than 40°C, the enzyme activity declined sharply. The enzyme showed maximum activity at pH 8.0, and in the alkaline region the activity declined rapidly. In the narrow range of pH 7.0–10, the activity was stable. *S*-HMGSH dehydrogenase from *P. variotii* NBRC 109023 had an optimum pH of 8.0; this value is the same as the optimal pH of *S*-HMGSH dehydrogenase from *Hansenula polymorpha* ([Demkiv et al. 2007]). On the other hand, *S*-HMGSH dehydrogenase from *P. variotii* NBRC 109023 had an optimum temperature of 40°C. This value is 10°C lower than that of the *H. polymorpha* enzyme ([Demkiv et al. 2007]).

The substrate specificity of *S*-HMGSH dehydrogenase was examined. Various substrates were added to the reaction mixture. As shown in Table 3*S*-HMGSH dehydrogenase from *P. variotii* NBRC 109023 has very high substrate specificity for formaldehyde; the only aldehyde or alcohol tested that served as a substrate other than formaldehyde was pyruvaldehyde. These properties are similar to those of the *Candida boidinii* enzyme ([Schutte et al. 1976]).

The effects of various compounds on enzyme activity were examined. Enzyme activity was enhanced by several divalent cations such as Mn^2+^, Ba^2+^ and Ca^2+^. On the other hand, Ni^2+^, Fe^3+^, Hg^2+^, PCMB and cuprizone completely inhibited activity. Inactivation of the enzyme by sulfhydryl reagents (Hg^2+^ and PCMB) indicated that the sulfhydryl group of the enzyme is essential for its catalytic activity. These inhibition results are similar to those of the enzyme from *C. boidinii*.

From the results obtained in this study, the properties of *S*-HMGSH from *P. variotii* NBRC 109023 are similar to those of the enzyme from *C. boidinii*; only the molecular weight of the enzyme and effects of metal ions on its activity differ. No publications have described the concentration of formaldehyde that *C. boidinii* can degrade. We cannot explain why *P. variotii* NBRC 109023 can degrade a high concentration of formaldehyde based on the results obtained. Comparison of the *S*-HMGSH dehydrogenase gene from *P. variotii* NBRC 109023 with that of other organisms, especially *C. boidinii*, is of great interest.

## Competing interests

The authors declare that they have no competing interests.
